# Potential of Vibroacoustic Therapy in Persons with Cerebral Palsy: An Advanced Narrative Review

**DOI:** 10.3390/ijerph16203940

**Published:** 2019-10-16

**Authors:** Jiří Kantor, Lucia Kantorová, Jana Marečková, Danping Peng, Zdeněk Vilímek

**Affiliations:** 1 Institute of Special Education Studies, Faculty of Education, Palacky University Olomouc, Žižkovo nám. 5, 77900 Olomouc, Czech Republic; lucia.kantorova@bahai.sk (L.K.); z.vilimek@gmail.com (Z.V.); 2 Department of Anthropology and Health Education, Faculty of Education, Palacky University Olomouc, Žižkovo nám. 5, 77900 Olomouc, Czech Republic; jana.mareckova@upol.cz; 3 Institute of Education and Social Studies, Faculty of Education, Palacky University Olomouc, Žižkovo nám. 5, 77900 Olomouc, Czech Republic; pengdanping2013@gmail.com

**Keywords:** children, adults, cerebral palsy, vibroacoustic therapy, spasticity, motor, movement, spasticity

## Abstract

Vibroacoustic therapy (VAT) is a treatment method that uses sinusoidal low-frequency sound and music. The purpose of this narrative review is to describe the effects of VAT on motor function in people with spastic cerebral palsy (CP) according to study design as well as providing information about the age of the participants, measurement tools, and sound frequencies that were used. The systematic search strategy based on the first two steps of a standard evidence-based approach were used: (1) formulation of a search question and (2) structured documented search including assessment of the relevance of abstracts and full texts to the search question and inclusion criteria. Out of 823 results of the search in 13 scholarly databases and 2 grey literature sources, 7 papers were relevant. Most of the relevant studies in children and adults presented significant improvement of motor function. According to the study design, only five experimental studies and two randomized controlled trial (RCT) studies were available. In the discussion, findings of this review are compared to other related methods that use mechanical vibrations without music. The authors recommend continuing to research the effects of VAT on motor function and spasticity in adolescents and young adults with spastic CP.

## 1. Introduction

Cerebral palsy (CP) is a complex functional disability, which, in medical literature, is defined as “a group of permanent disorders of the development of movement and posture, causing activity limitation, that are attributed to non-progressive disturbances that occurred in the developing fetal or infant brain. The motor disorders of cerebral palsy are often accompanied by disturbances of sensation, perception, cognition, communication, and behavior, by epilepsy, and by secondary musculoskeletal problems” [1] (p. 572). CP is one of the most common childhood disabilities [2], with prevalence rates between 2–3.5 per 1000 live births reported [3]. 

Spasticity is the most prevalent form of CP, with increased muscle tone and typical spastic symptoms ranging from mild neurological deficits to severe impairment. Spasticity is composed of neural and secondary non-neural components, including muscle structure or connective tissues [4]. It affects over an estimated 12 million people worldwide, and about 80% of people with CP have varying degrees of spasticity [5]. Spasticity is linked with limited range of motion, which is a measurement of movement around a joint. Other symptoms are stiffness or tightness of the muscles, muscle and joint deformities, muscle fatigue, inhibition of longitudinal muscle growth, and so on [6]. The level of severity is categorized by the Gross Motor Function Classification System (GMFCS), with values of I–V. 

As for the development of motor functions, age is an adverse factor for a high percentage of people with CP. According to Rosenbaum et al. [7], children in all severity levels achieve most of their potential function early; by age seven, function generally begins to level off. Tendencies of motor function to decline in adulthood have been mentioned by other studies as well [8]. Possible explanations proposed for motor decline are increased body size; decreased activity; changes in spinal alignment [9], muscle flexibility, strength, and endurance; and increased spasticity, arthritis, falls and fractures, and pain or fatigue [10]. Ritzman, Stark, and Krause [4] (p. 1620) state the following in relation to therapeutic possibilities and age: “In the last few decades, scientific debate in the field of neurorehabilitation has consistently concluded that training regimes commonly achieve higher efficiency in children compared to adolescent patient groups.” 

The treatment methods of spastic CP include physiotherapy [11], surgery, and drug administration, such as botulinum toxin A [12]. There is also a growing scientific evidence of the effects of vibration therapy—a non-invasive and safe training modality that uses mechanical oscillations. Growing research on vibration therapy and CP population nowadays includes a range of randomized controlled trial (RCT) studies as well as systematic reviews [4,13,14].

So far, the discussions about different possible theories about therapeutic mechanisms of vibration therapy and CP are still ongoing. Basically, vibrations influence neuromuscular structures in the body [4], with somatosensory perception playing a key role. The theory of neuromodulation explains the therapeutic effect via stimulation of alpha-motor neurons; however, vibrations also stimulate mechanoreceptors and induce neuroplasticity via somatosensory/motor pathways and (concerning whole body vibrations, WBV) increase neural drive to the muscles with resulting increase in muscle mass and strength [15]. Vibration determinants refer to the frequency (number of complete cycles per second, 5–200 Hz), amplitude (vertical displacement 0.5–10 mm), and type of vibration therapy (VT; sinusoidal vertical and side-alternating) [4]. The vibration can be provided as focal vibration to a specific muscle/tendon or as whole-body vibration. Low-frequency stimulation can be derived from mechanical vibrations (e.g., through an oscillating platform or membrane) as well as from sound vibrations. To conclude briefly, there is more than one strategy of vibrations application for therapeutic purposes. 

Vibroacoustic therapy (VAT) is a relatively new method in the area of vibration therapy, as its foundations were laid in the 2nd half of the 20th century. It uses low-frequency sound (under 100 Hz) in the audible range to produce mechanical vibrations, which differs from methods using only infrasonic frequencies (under 20 Hz) that are not audible to the human ear [16]. In VAT, low-frequency vibrations are derived from technologically modified sound waves (not from mechanical vibrations). Another distinctive characteristic of VAT is using low-frequency vibrations together with music listening (comparing, for example, WBV that are typically applied while standing on an oscillating platform that displaces the individual and alters the gravitational forces of the body) [17]. 

The roots of VAT are connected to a Nordic neurologist and pedagogue, Skille, and his experiments using deep vibrations in people with multiple disabilities. He defined VAT as “using sinusoidal low-frequency sound in 30–120 Hz range complemented by music for therapeutic purposes” [18] (p. 36). In the 1990s, the physioacoustic method based on scanning the body with sinusoidal sound between 27–113 Hz and specially selected music listening was developed by Lehikoinen [19]. Other examples of vibroacoustic devices include Eakin’s multiple designs of Somatron Corporation first released in 1985 (Tampa, Florida, USA), the music vibration table (MVT) designed by Chesky in the late 1980s (there is no indication that MVT is in manufacture) [20], the HealBED first released in 1990–1991 (HealBED, Haapsalu, Estonia) [21], Multivib products (that use a mattress or a cushion with embedded vibration speakers, Multivib as, Trondheim, Norway) [22], Vibrobed developed in 2018 by Vilímek and Švarc (Vibroacoustic Brothers, Olomouc, Czech Republic) [23], the Relaxation Lounge V1 and V2 of Nex Neuro Vibro-Acoustic Therapy [24], Sonobed^™^ from Heritage Medical Associates, P.C., Nashville, Tennessee, developed in 1993, and the Vibroacoustic Therapy System VTS1000 of Sound Oasis Company (Marblehead, Massachusetts, USA) [25] or Subpac (Subpac Inc, Toronto, Canada) [26].

Based on the type of low-sinusoidal stimulation, selective low-frequency (SLF) and full-frequency music (FFM) may be differentiated [27]. SLF uses specific low frequencies for vibroacoustic stimulation, such as Skille’s equipment, whereas FFM uses “a single sound source and plays music using a wide range of frequencies” (such as quantified mechanical vibration design of Chesky´s music vibration table) [27] (p. 112). As for the selective low-frequency stimulation, an important issue is connected to the selection of suitable frequencies [28]. Although Skille [29] formulated a set of seven frequencies, this set is based merely on practical experiences, and there is a lack of theoretical support. One of the exceptions is the theory of thalamic frequency of 40 Hz [30]. 

**Figure 1:**
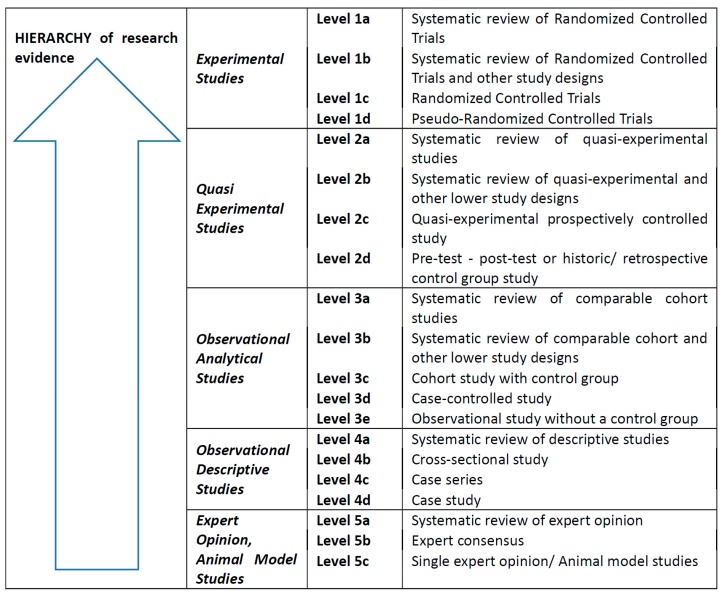
Levels of evidence in quantitative research [34].

VAT is traditionally indicated for use in spastic CP and other brain problems, as it helps to enhance physiotherapeutic intervention and improves motor and brain functions [31] (e.g., through circuit connectivity through oscillatory coherence). VAT is also beneficial for its effects on quality of life, well-being, and stress management [28]. Concerning spinal cord and brain injuries, VAT can lead to changes in the levels of spasticity, pain, physical discomfort, general health condition, fatigue, and anxiety [32]. Although it is possible to find other information on the effects of VAT in normalisation of muscle tone in people with spastic CP [18,31], systematically led reviews on research about its effects (comparing some other approaches in vibration therapy) are lacking. However, such evidence would be of benefit to the scientific background of VAT since current science and practice is increasingly applying the evidence-based healthcare (EBHC) concept: “evidence-based medicine requires skills of literature retrieval, critical appraisal, and information synthesis. It also requires judgment of the applicability of evidence to the patient at hand and systematic approaches to make decisions when direct evidence is not available“ [33]. The most robust evidence for practice is, according to this concept, found in the results of secondary research represented by systematic reviews using a meta-analysis or meta-aggregation. The hierarchy of primary research evidence starts with randomized controlled trials and ends with expert opinion (see Figure 1). The authors of this paper have, therefore, focused on the use of these elements of the EBHC concept to identify, classify, and describe the design of existing studies on the effects of VAT on motor functions in people with CP.

## 2. Materials and Methods

The purpose of this review is to identify relevant published information (from research studies in scientific databases and grey literature sources) on the effects of VAT on motor function in people with spastic CP according to the study design. Additional aims include the identification of:

- the age of the included people with CP;
- what measurement tools have been implemented in research on effects of VAT on motor function of people with spastic CP; and
- what low-frequency or types of low-sinusoidal stimulations have been used in research on the effects of VAT on motor function of people with spastic CP.

To meet the objective, a search question (SQ) was formulated using the problem–intervention–comparison–outcome (P–I–(C–not applicable)–O) components with additional synonymous and related terms (Table 1): What is the research evidence on the therapeutic effects of vibroacoustic therapy (I) on motor function (O) in people with spastic cerebral palsy (P)?

A standard search strategy was applied as recommended by evidence-based healthcare methodology [12]. The primary search terms, as an input to the development of the search strategy, were P, cerebral palsy; I, vibroacoustic therapy; C, not applicable; and O, motor function. To increase the search sensitivity, synonyms and related terms were added to the primary search terms using the Boolean operator “OR” (Table 1). Roget´s 21st Century Thesaurus was used to formulate synonyms and similar terms. To increase the search specificity of partial results for the P–I–O, components were connected using the Boolean operator “AND” (Table 1). The following strategy was used: title/abstract; search period not limited (to increase search sensitivity); no language restrictions. The search was carried out in May 2019, and the Cochrane Library, Joanna Briggs Institute Library (JBI), and Prospero databases were added in September 2019.

The following databases were searched:

- PubMed,
- MEDLINE complete,
- Bibliographia Medica Čechoslovaca (the Medvik interface),
- EBSCO host,
- EBSCO discovery,
- ERIC,
- Wiley Online Library,
- EBM Reviews,
- ProQuest,
- Scopus,
- CINAHL Plus (with Full Text),
- Cochrane Library,
- JBI
- Prospero,
- MedNar, and
- Google Scholar.

Grey literature resources from web search engines MedNar and Google Scholar were included based on the Joanna Briggs Institute (JBI) standard recommendations to increase the thoroughness of search results [34]. When searching in the e-sources, the specifics of their search engines were taken into consideration (Table 2). Moreover, the search strategy included an additional manual search of references of relevant studies, which diminished the risk of overlooking relevant studies or not finding them due to inadequate indexing. 

Inclusion criteria: only primary research studies relevant to the search question (i.e., the P–I–O components) were included in the narrative review. Only studies that involved participants with spasticity symptoms were included. The minimum JBI level of evidence 4d was used for quantitative evidence (Figure 1). By the term vibroacoustic therapy we mean the sound-induced, low-frequency vibrations (not mechanical vibrations) mixed with music listening [8]. 

Exclusion criteria: 1. application of similar therapeutic methods without technologically modified low-sinusoidal sound (e.g., monochords, acoustic vibration beds, or methods using mechanical vibrations); 2. application of low-frequency sound in multimodal therapeutic settings (where it is not possible to evaluate the effect of sinusoidal sound separately from other stimuli); 3. all types of reviews, preconference abstracts, bachelor and diploma theses; and 4. research with participants with extrapyramidal syndromes and central hypotony and no spasticity.

The selection and analysis of studies was based on removing duplicates and assessing the relevance of abstracts to the search question and its components represented by P–I–O and the inclusion criteria. For relevant abstracts, full texts of articles were retrieved and once again carefully assessed for relevance to the search question. Full texts of the relevant studies were sorted and divided into categories based on the type of study design. Categories according to the JBI levels of evidence in quantitative research (Figure 1) were used for the analysis of advanced search results. In case that full texts of papers included information on the methodological design, the evidence level by JBI was determined based on that information. In case the design of the study was not mentioned in the full text, the authors determined it based on an analysis of the research methodology and other information provided in the study. 

## 3. Results

**Figure 2:**
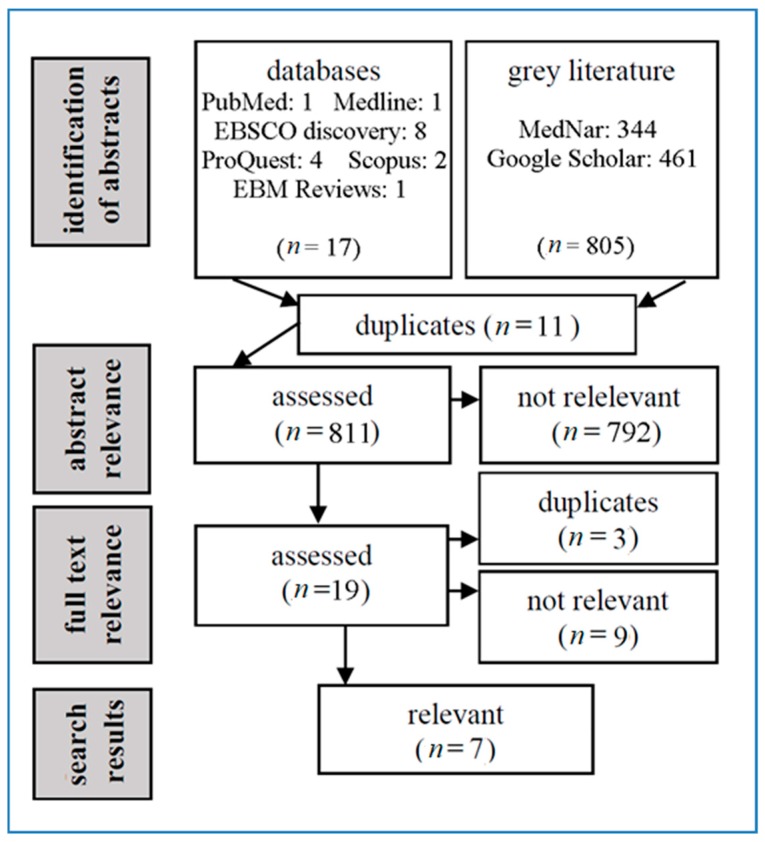
Flow diagram of the literature search (searches in databases with zero results are not listed in the flow diagram).

For the search question, 823 resources were identified (18 abstracts in scholarly databases and 805 in grey literature sources). After assessment of their relevance to the inclusion and exclusion criteria (see the Methods section), 7 of them were classified as relevant. Five of them were full texts of studies indexed in scholarly databases, and the remaining 2 (dissertations) were included in the grey literature. The search process is shown in Figure 2.

Since the dissertation “The effects of vibroacoustic therapy on clinical and non-clinical populations“ [18] presented a series of 5 studies, the relevance of each was assessed separately. Chapters 4 and 5 were found relevant [35,36]. These chapters were also published in “Music vibration and health” [37]. The dissertation ”The effect of 40 Hz sound wave vibration on spasticity and motor functions in children with cerebral palsy“ of Katušic [38] was evaluated as a duplication as it contained the research published in the RCT study of Katusic, Alimovic, and Mejaski-Bosnjak [39]. There were also 2 papers in Chinese language [40,41] that were translated into English and included in the relevant search results.

The analysis of the methodological design of relevant studies (see below) showed that secondary research studies (systematic reviews) were completely lacking; however, 2 contributions with RCT design [39,42] and 5 contributions with experimental study design [35,36,40,41,43] were available. The following sections present in detail available information on the methodological design of 7 studies, the effects of VAT on movement and spasticity, instruments used for measuring, ages of participants, and frequencies used in the research. Since age is an important factor influencing motor functions in persons with CP [10], the studies were divided, for the purposes of this paper, into two sections of those concerning children with CP (4 studies summarised in Table 3, Table 4, Table 5 and Table 6) and those exploring the adult population with CP (3 studies summarised in Table 7, Table 8 and Table 9). 

### 3.1. Results of Relevant Studies Applying VAT in Children with CP

The 4 studies (Table 3, Table 4, Table 5 and Table 6) applying VAT in children with CP (age 1–8), including one RCT study [39], all stated a statistically significant improvement in children’s range of motion (motor function). The 3 other studies used a pretest–post-test quasiexperimental design [40,41,43]. Moreover, two of the studies also focused on investigating spasticity and found that VAT significantly reduced muscle tone in CP [39,40]. One study found statistically significant differences in the classification (GMFCS, see below) levels of children before and after intervention [39].

One study implemented a new instrument (developed for this study) for measuring range of motion [41]. The remaining 3 studies on children with CP used standardized methods: Gross Motor Function Measurement (GMFM) for measuring range of motion, Modified Modified Ashworth Scale (MMAS) for measuring muscle tone, and Gross Motor Function Classification System (GMFCS) to classify children with CP according to their motor function.

Furthermore, the studies have experimented with applying various frequencies of sinusoidal sound. In two studies [39,43], a frequency 40 Hz was used as recommended by Skille [11]. The team of Liu [40] decided to use a frequency 60 Hz, and another study by Liu [41] stated using 16–150 Hz frequencies.

### 3.2. Results of Studies Applying VAT in Adults with CP

Three relevant studies (Table 7, Table 8 and Table 9) investigating the effects of VAT on motor function in adults with CP (age 24–77) all showed a positive effect of VAT, one being RCT [42] and the other two a pretest–post-test quasiexperimental design [35,36]. Although all participants showed improvement of their motor functions in the study by Kvam [42] on 12 adults with CP, only hand–eye coordination results were statistically significant. In this study, most participants were better in a drawing test, and improvement in fine movements was quite significant. The group receiving VAT was more sure of their desire to continue (with statistical significance) and more positive on the effect of the treatment. The other two studies [35,36] on a total of 20 adults with CP showed a statistically significant improvement of motor function in the VAT group when compared to the group receiving a placebo. None of these studies explored effects on spasticity.

All three studies used nonstandardized measurement instruments. Kvam [42] used gross and fine movement tests and drawing/writing tests (Swedish test by Bille). Studies by Wigram [35,36] measured changes in range of motion in specifically given areas with a marker and a standard tape measure.

Wigram [35,36] used a sinusoidal sound of 44 Hz frequency, which was very close to the 40 Hz used by Katusic mentioned above. The study by Kvam [42] stated using a range between 40–80 Hz frequency.

## 4. Discussion

This section is structured according to main and additional goals of this paper with the aim to compare findings of this advanced review with findings of systematic reviews and RCT studies from the broader area of vibration therapies (methods using mechanical vibrations), namely WBV. These studies were found in Cochrane Library and PubMed databases.

There were two randomized controlled trials (RCTs): one in adults, one in children, and five pretest–post-test quasiexperimental studies on VAT in people with CP. Out of the two RCT studies found, one was a single-blind study [39], one double-blind [42], and there were no triple-blind studies in this area. Systematic reviews focused on VAT and CP have not been realized so far, although some related methods using mechanical vibrations have this level of evidence available, for example, in the case of WBV [13,43] or vibration therapy generally [4]. According to the authors from our team, further research in VAT is needed to achieve more robust scientific evidence in the future; specifically required are RCT studies, which provide a higher level of evidence in quantitative research than experimental studies [33]. The systematization of information concerning methodological designs of studies may help in the future creation of systematic review protocols focused on exploring the effects of VAT on motor functions of people with CP.

Published studies show that VAT may increase motor function in both children and adults with CP. All seven studies presented an improvement of motor function in participants, six of them with statistical significance [35,36,39,40,41,43]. Furthermore, three of these studies found that VAT reduced spasticity in people with spastic CP [39,40,41]. These findings are consistent with findings of systematic reviews on WBV [13,44], proving that low vibrations can improve movement and spasticity in people with CP. 

VAT studies focused on adult populations included participants aged 24–77 years old, and VAT studies of child populations included participants aged 1–8 years old. Studies on adolescents and young adults (ages 9–23) were, therefore, completely missing. This age category has, however, been explored in studies dealing with WBV that brought positive results in, for example, a study done by Krause et al. (ages 4–22) [45] or Tupimai (ages 6–18) [46]. It can, therefore, be assumed that it may be possible to observe positive effects of VAT also in the age category of adolescents and young adults with CP, although these results may not be as significant in terms of their healing potential as in the case of children with CP. “Although VT (vibration therapy) demonstrably has a positive and age-independent influence on neuromuscular, functional, and structural factors associated with the disease-related deficits in patients with CP, we expect that VT will be particularly efficient in children, due to their advantage of neuroplasticity, and that VT may initiate long-term developmental effects that may persist into adulthood” [4] (p. 1621). 

As for the measurement tools, two studies on VAT [39,43] used GMFM-88 (Gross Motor Function Measurement) to measure motor function and GMFCS (Gross Motor Function Classification System) for classification according to motor function; three studies on VAT [39,40,41] used MMAS (Modified Modified Ashworth Scale) to measure spasticity; one study on VAT [42] used Bille´s test of motor function (FBH); two by one author used an original range of motion measurements [35,36]; and two by one author [40,41] used measuring of range of motion of some body parts with a tape measure. Only research studies in children used internationally recognized measurement tools. The benefit of these standardized tools is their easy interpretation for researchers worldwide and the possibility of enhancing the scientific robustness of such studies. Four studies used original measurement methods that may be hard to exactly replicate, even with a thorough description, which is sometimes lacking. 

Regarding frequencies of sinusoidal sound, two studies used a 40 Hz frequency [39,43]; two studies used 44 Hz [35,36]; another one used 60 Hz [40]; one used a nonspecified range of 40–80 Hz [42]; and one stated a broad range of 16–150 Hz [41]. These findings correlate with Skille’s conclusions [29] since he associates 40 and 60 Hz frequencies with the treatment of spasticity. Authors of this paper have not found any studies aiming to determine the effects of various frequencies or of various types of low-sinusoidal stimulation on movement or spasticity in people with CP. Also, findings from systematic reviews from the area of vibration therapy are inconclusive in this issue. For example, Ritzmann, Stark, and Krause [4] write: “Among the different intervention protocols, the analysis revealed no apparent dosage-dependency of VT. The great variation in VT protocols makes it difficult to highlight a clear favorite setting with efficiency beyond the others. Vibration has been applied at different levels: 5–50 Hz for WBV and reaching peak frequencies for focal vibration at 60–200 Hz” (p. 1620). There is a possibility that different sound frequencies or different types of low-sinusoidal stimulation could have different therapeutic outcomes; however, this must be explored in the future.

### 4.1. Recommendations for Future Research

Future researchers could, when designing research in this area, take the following into consideration.

- There have been few RCTs, and, specifically, double-blind and triple-blind studies are completely missing.
- Effects of VAT on adolescents and young adults with CP have not been explored yet.
- Using standardized measurement tools enables comparisons of results.
- It is advisable to include measuring spasticity (using MMAS).
- There is little knowledge on the possibly of different therapeutic outcomes of various frequencies or types of low-sinusoidal stimulation.
- There are other topics not addressed sufficiently in this review that would be worthy of further research (e.g., exploring the mechanisms of how VAT influences neuromuscular functions).

If future researchers could broaden primary research focused on the application of VAT in people with spastic CP, it would then be possible to include such data (e.g., in a meta-analysis) in the creation of systematic reviews and guidelines for the application of VAT. Modern approaches of evidence-based practice (according to Cochrane or JBI methodology) lead experts towards using evidence from studies with more robust levels of evidence that are listed higher in the hierarchy of studies. Based on this assumption, it would be beneficial to create systematic reviews in order to prepare guidelines for VAT practice in the future. We, therefore, recommend the expert community to continue realizing further research focusing on the effects of VAT in people with CP, with priority on RCT study design. 

### 4.2. Reflection of the Review

Some procedures of the standard evidence-based approach were used for this advanced narrative review, namely the formulation of a search question; a structured, documented search; assessment of the relevance of abstracts and full texts to the search question and inclusion criteria; searches in a large number of relevant databases; inclusion of studies in different languages and their translations (e.g., Chinese); and so on. Since this is not a systematic review, the studies were not assessed by two or more independent evaluators, the methodological quality of contributions was not critically appraised, and a meta-analysis of data was not included.

The scale of the researched issue had also been reflected. In this paper, only studies about VAT as defined in the first section were searched. This means that we searched for studies based on sound-induced vibrations (not mechanical vibrations) because academic literature differs methods in this area according to the source and type of vibrations [47]. Furthermore, we searched for studies using low-frequency sound in combination with music listening. This is another difference between VAT as defined by Skille [8] or Lehikoinen [19] and typical practice of many therapists using methods based on mechanical vibrations without music. The fact that music itself can significantly impact motion and spasticity [48] cannot be ignored. Especially, modern VAT devices [23] that are based on an interaction of vibrations and music bring a new aesthetic dimension for listening to music and, most importantly, a potentially new combination of known mechanisms impacting somatic and mental (emotional) functions of the human body. Therefore, only methods of vibroacoustics as described above were included in the search of this review, which were then, in discussion, compared with other related methods.

## 5. Conclusions

The results show that VAT could potentially improve motor function in children and adults with spastic CP. Although this conclusion has been confirmed by most of the found studies, primary studies with highly robust evidence (RCT) are few, and evidence on the level of secondary research (SR) is completely lacking. Partially, this deficit is possible to compensate by reflecting findings on VAT in people with CP in the context of similar findings concerning related methods from the area of vibration therapies (based on mechanical vibrations). The authors recommend continuing to research the effects of VAT on motor function and spasticity in people with spastic CP (with priority of RCT study design), to focus on adolescents and young adults, and to compare the effects of various frequencies and types of low-sinusoidal sound stimulation. Furthermore, during future research in this area, standardized methods of measurement already used in previous studies are preferred.

## Figures and Tables

**Table 1:** Synonymous and related terms for the P–I–O components.

**Primary search terms**	
P	Spastic cerebral palsy
I	Vibroacoustic therapy
C	NA
O	Motor function
**Primary search terms + synonyms + related terms**	
P	“cerebral palsy“ OR “spastic paralysis“ OR quadriparesis OR diparesis OR hemiparesis
I	“vibroacoustic therapy“ OR “vibroacoustic music“ OR “vibroacoustic sound“ OR “somatosensory music therapy“ OR physioacoustic OR “physio acoustic sound” OR somatron OR “low-frequency sound stimulation” OR “vibrotactile stimulation” OR “music vibration table”
C	NA
O	“motor function” OR “range of motion“ OR “movement AND spasticity”

P—problem; I—intervention; C—comparison; O—outcome; NA—not applicable.

**Table 2:** Search results.

Database	Results for P (*n*)	Results for I (*n*)	Results for O (*n*)	Search Results (*n*)
PubMed	31,713	333	265,208	1
Medline	25,671	292	310,806	1
BMČ (Medvik)	196	1	1572	0
EBSCO discovery	112,674	1547	2,905,734	8
ERIC	2980	255,974	1,673,393	0
Wiley Online Library	9980	0	2,300,473	0
EBM Reviews	4962	39	37,242	1
ProQuest	99,370	652	2,756,038	4
Scopus	59,723	2407	2,649,382	2
CINAHL Plus	8998	40	60,427	0
Cochrane Library	77	45	99	0
JBI	38	484	2	1
Prospero	598	0	92	0
MedNar *	1388	313	2385	344
Google Scholar *	cca 550,000	cca 8990	cca 5,560,000	461

* Results for primary keywords, not the synonyms.

**Table 3:** Studies on children with CP—Katusic, Alimovic, and Mejaski-Bosnjak [39].

Research Design	Randomized Controlled Trial, Single-Blinded
JBI level of evidence	1c
Objective(s) stated in the study	To determine the effects of VAT on spasticity and motor function in children with CP (age 4–6) undergoing physiotherapy compared to physiotherapy alone
Sample	89 children (age 4–6) with spastic CP from a daycare center for rehabilitation were randomized into conventional therapy or conventional + VAT.
Randomization	Stratified according to GMFCS level
Intervention	Experimental group: VAT (on top of conventional therapy) twice a week for 12 weeks (40 Hz with sinusoidal amplitude variations −6.8 s between peaks). Control group: physiotherapy 3 times a week for 40 min.
Measurements	Pre- and post-12-week period MMAS spasticity, GMFM-88 by blinded evaluators. Statistical methods: non-parametric Wilcoxon matched pairs test and Mann–Whitney U test.
Results	The 12-week-change MMAS total score showed that spasticity level decreased more in the VAT group (*p* < 0.001). GMFM-88 significantly improved more in the VAT group (*p* < 0.001).

Abbreviations used: JBI—Joanne Briggs Institute, CP—cerebral palsy, VAT—vibroacoustic therapy, GMFM-88—Gross Motor Function Measure, GMFCS—Gross Motor Function Classification System, and MMAS—Modifed Modified Ashworth Scale.

**Table 4:** Studies on children with CP—Liu, Zhang, and Zhao [40].

Research Design	Quasi-Experimental Prospective Study
JBI level of evidence	2c
Objective(s) stated in the study	To determine the effects of VAT on muscle tension and range of motion in children with spastic CP (age 1–6) compared with physiotherapy and placebo.
Sample	90 children (age 1–6) from Nanhai Affiliated Maternity and Children’s Hospital of Guangzhou University of Traditional Chinese Medicine allocated equally into three groups: conventional therapy, placebo, and VAT. The study does not specify how participants were allocated into groups. There was no significant difference between the groups for gender, age, and for some of the muscle tone and range of motion measurements (*p* > 0.05).
Intervention	Conventional therapy group—physical therapy, massage, and Chinese herb bath (once a day for 20 d). Experimental group—conventional therapy + VAT (60 Hz with Jiao music). Control group—conventional therapy + Jiao music. Interventions for both groups: 30 min a day for 20 d.
Measurements	An average of 3 measurements of muscle tone and range of motion (before, midway, and after treatment) were used, muscle tone assessed by MMAS, and statistical methods used included matching *t* test and variance.
Results	There was no statistically significant difference for the conventional group after 20 d of treatment. Listening (placebo) group had a decrease in muscle tone (*p* < 0.05). In the VAT group, the range of motion of hips, knees, and ankles improved, and muscle tone decreased (*p* < 0.05). 89 children (age 4–6) with spastic CP from a daycare center for rehabilitation were randomized into conventional therapy or conventional + VAT.

Abbreviations used: JBI—Joanne Briggs Institute, CP—cerebral palsy, VAT—vibroacoustic therapy, and MMAS—Modifed Modified Ashworth Scale.

**Table 5:** Studies on children with CP—Katusic and Mejaski-Bosnjak [43].

Research Design	Pretest–Post-Test Quasiexperimental Study
JBI level of evidence	2d
Objective(s) stated in the study	To determine the effects of VAT on spasticity and motor function in children with spastic CP (age 3–4)
Sample	13 children (age 3–4) from a daycare center for rehabilitation.
Intervention	VAT once a week for 12 weeks, 20 min (40 Hz sine wave with sinusoidal amplitude variation, 6.8 s between peaks).
Measurements	Pre- and post-12 week treatment GMFM-88, GMFCS standardised assessments, non-parametric Wilcoxon matched pairs test and Pearson c 2-test.
Results	Significant improvement in motor function. There was a significant improvement in total GMFM score (*z* = 3.17, *p* = 0.00), as well as on dimension A (lying and rolling, *z* = 3.05, *p* = 0.00) and dimension B (sitting, *z* = 2.80, *p* = 0.00). There was a significant difference between GMFCS levels of children before and after intervention (df = 6, *p* = 0.00).

Abbreviations used: JBI—Joanne Briggs Institute, CP—cerebral palsy, VAT—vibroacoustic therapy, GMFM-88—Gross Motor Function Measure, and GMFCS—Gross Motor Function Classification System.

**Table 6:** Studies on children with CP—Liu et al. [41].

Research Design	Pretest–Post-Test Quasiexperimental Study
JBI level of evidence	2d
Objective(s) stated in the study	To determine effects of VAT on muscle tension and range of motion in children with spastic CP (age 2–8)
Sample	36 children (2–8 years) with spastic cerebral palsy in outpatient and hospitalized care at children´s rehabilitation hospital clinic.
Intervention	Application of vibroacoustic therapy (16–150 Hz) with Jiao music 30 min each time. No mention of length of therapy.
Measurements	Measuring range of motion, muscle tension: MMAS. Statistical methods used: *t* test.
Results	After VAT, the adductor angle, popliteal fossa angle, food dorsiflexion angle, and muscle tone improved (*p* < 0.05).

Abbreviations used: JBI—Joanne Briggs Institute, CP—cerebral palsy, VAT—vibroacoustic therapy, and MMAS—Modifed Modified Ashworth Scale.

**Table 7:** Studies on adults with CP—Kvam [42].

Research Design	Double-Blind Randomized Controlled Trial
**JBI level of evidence**	1c
**Objective(s) stated in the study**	To determine effects of VAT in adults with CP (age 27–48) on gross and fine movements compared to placebo
**Sample**	From 1 sheltered workshop of 14 workers, 12 had CP and were included.
**Randomization**	Stratified randomization of sample size 12: 6 pairs were created based on similarities in age, level of communication, physical functioning, and level of independence. One member of each pair was randomly selected for the experimental group.
**Intervention**	Experimental group: application of VAT (40–80 Hz) twice weekly for 9 weeks. Control group: application of music only, no vibrations, twice weekly for 9 weeks.
**Measurements**	Videotapes of gross and fine movements and drawing/writing tests (Swedish test by Bille) blind evaluation by 4 assessors, AND post-trial interviews. Statistical methods used: non-parametric one-tailed Wilcoxon signed rank test for matched pairs, inter-rater reliability of the test was 90% and of the drawing test 92%.
**Results**	All experimental participants showed greater improvement after treatment in motor test, and most participants were also better in drawing tests than those in the control group. In the area of hand–eye coordination, there was a statistically significant improvement (*p* = 0.034), and improvement in fine movements was quite significant (*p* = 0.069). In the final interviews with participants, the experimental group was more positive as to the effect of the treatment (*p* = 0.054) and more certain of their desire to continue (statistically significant, *p* = 0.034). No participant results were worse after the treatment.

Abbreviations used: JBI—Joanne Briggs Institute, CP—cerebral palsy, and VAT—vibroacoustic therapy.

**Table 8:** Studies on adults with CP—Wigram, chapter 4 [35].

Research Design	Pretest–Post-Test Quasiexperimental Study (Included Subjects Studied with a Single Blind Evaluation)
JBI level of evidence	2d
Objective(s) stated in the study	To determine effects of VAT on range of motion in adults (age 28–77) with CP and high muscle tone compared with placebo (only music)
Sample	10 residents of a large mental hospital took part in the trials (28–77 years), with severe disorder with spastic cerebral palsy, with high muscle tone.
Intervention	All 10 subjects received 6 sessions of 30 min VAT (44 Hz) as well as 6 only music (no vibrations) sessions, randomly ordered, 2 times a week over 6 weeks.
Measurements	Blind evaluation of spinal mobility and limb flexion and extension using tape measures of range of movement before and after each session. Statistics: Wilcoxon matched pairs signed rank test.
Results	VAT treatment showed significantly improved range of movement than placebo (no vibrations), *p* = 0.0051.

Abbreviations used: JBI—Joanne Briggs Institute, CP—cerebral palsy, and VAT—vibroacoustic therapy.

**Table 9:** Studies on adults with CP—Wigram, chapter 5 [36].

Research Design	Pretest–Post-Test Quasiexperimental Study
**JBI level of evidence**	2d
**Objective(s) stated in the study**	To determine effects of VAT on range of motion in adults (age 24–68) with high muscle tone compared to placebo
**Sample**	10 participants were randomly chosen from a group of 27 residents of a large mental hospital (24–68 years) all with high muscle tone who were included in a different part of the same study.
**Intervention**	Three 30 min VAT sessions using 44 Hz and three music (placebo) sessions in each of the 10 participants administered blindly.
**Measurements**	Range of motion, extension of arms and legs in centimeters measured before and after each treatment, statistics: Wilcoxon matched pairs signed rank test.
**Results**	All 10 participants had improved motor function after VAT compared with a placebo (only music) with a significant difference *p* = 0.0051.

Abbreviations used: JBI—Joanne Briggs Institute, CP—cerebral palsy, and VAT—vibroacoustic therapy.

## Note

Although triple-blinded studies are recommended for clinical research [24], the design of double-blinded studies seems sufficient for VAT research. There is a very low probability that a person offering intervention could influence the result of the study.
